# Implementation of music in colorectal perioperative standard care—barriers and facilitators among patients and healthcare professionals

**DOI:** 10.1111/codi.16102

**Published:** 2022-04-06

**Authors:** Ellaha Kakar, Oddeke van Ruler, Bram van Straten, Bas Hoogteijling, Eelco J. R. de Graaf, Erwin Ista, Johan F. Lange, Johannes Jeekel, Markus Klimek

**Affiliations:** ^1^ Department of Surgery and Intensive Care Unit Erasmus MC University Medical Centre Rotterdam The Netherlands; ^2^ 10192 Department of Surgery IJsselland Hospital Capelle aan den IJssel The Netherlands; ^3^ Erasmus MC University Medical Centre Rotterdam The Netherlands; ^4^ 10192 Department of Anesthesiology IJsselland Hospital Capelle aan den IJssel The Netherlands; ^5^ Department of Internal Medicine Section Nursing Science University Medical Centre Erasmus MC Rotterdam The Netherlands; ^6^ Department of Surgery University Medical Centre Erasmus MC Rotterdam The Netherlands; ^7^ Department of Anesthesiology University Medical Centre Erasmus MC Rotterdam The Netherlands

**Keywords:** implementation, music, surgery

## Abstract

**Aim:**

Perioperative anxiety and pain are still prevalent among patients undergoing surgery. Inflammatory bowel disease and colorectal cancer patients are known to have higher anxiety rates than the general population. Perioperatively applied music intervention has been proven to be effective in reducing perioperative anxiety and pain, resulting in a decrease of intra‐operative sedative use, postoperative opioid requirement and neurohormonal stress response. IMPROVE evaluates the adherence to music intervention in colorectal perioperative standard care during systematic implementation.

**Method:**

The Consolidated Framework for Implementation Research (CFIR) was used for implementation in three steps. This study addresses the first step in which barriers and facilitators for implementing perioperative music were identified by surveying patients who underwent colorectal surgery and healthcare professionals involved in perioperative care. Also, perioperative anxiety scores were assessed and data on perioperative pain was collected from the patients’ medical records.

**Results:**

Fifty patients and 69 professionals (response rate 68.3%) were surveyed. For patients, all domains of the CFIR were facilitating implementation. The median reported preoperative and postoperative anxiety scores were 4.5 (1.0–7.0) and 3.0 (1.0–5.75) respectively. The median postoperative pain score on the first postoperative day was 2.8 (2.0–3.7). Also, for professionals most domains were facilitating, except for some factors related to work climate and culture among nurses.

**Conclusions:**

In this study it was identified that facilitating factors for implementing music in standard perioperative care were more prominent in both patients and healthcare professionals and therefore successful implementation is probable. Also, this study provides a guideline for assessing facilitators and barriers in other settings.

**What does this paper add to the literature?**

Perioperative anxiety and pain, which are significantly improved by music, are still prevalent among inflammatory bowel disease and colorectal cancer patients. However, implementation of new interventions is challenging. This study describes essential barriers and facilitators for successful implementation of music and provides a framework to assess them in other settings.

## INTRODUCTION

Preoperative anxiety is prevalent among surgical patients, described in 75% of the patients facing surgery [1, 2]. Inflammatory bowel disease (IBD) and cancer patients are known to have higher anxiety rates than the general population [3, 4, 5]. Many of these patients undergo major surgical procedures, which cause additional psychological stressors [6, 7]. Anxiety can cause resistance, which is defined as a range of negative emotions about the surgical procedures [8]. Resistance can affect the perioperative course, including more difficult induction and maintenance of anaesthesia, higher postoperative analgesic requirement and less satisfaction of the patient with the perioperative experience [8, 9, 10, 11, 12, 13, 14, 15]. Preoperative anxiety and pain are also predictive factors for the intensity of postoperative pain [13]. Despite many efforts to optimize the treatment of acute postoperative pain, still 40%–65% of the patients experience moderate to severe postoperative pain [14, 15]. Postoperative pain is related to increased morbidity, development of chronic postoperative pain, functional and quality of life impairment, poor postoperative recovery, prolonged opioid use and increased medical costs [16]. Pharmacological agents, such as opioids and benzodiazepines, are widely used interventions as treatment for anxiety and pain even though their side‐effects and risks such as respiratory depression and dependency are well known [17, 18, 19]. Benzodiazepine usage often leads to sedation‐related side‐effects such as drowsiness, dizziness and light‐headedness leading to falls and other accidents [20, 21, 22, 23, 24].

Therefore, non‐pharmacological interventions should be considered in the perioperative standard of care of patients undergoing surgical procedures. The literature shows that perioperatively applied (recorded) music intervention can reduce preoperative anxiety and postoperative pain, resulting in a decrease of intra‐operative sedative and postoperative opioid requirement [25, 26, 27]. Its effectiveness was also observed in the neuro‐endocrine stress response, resulting in lower levels of postoperative cortisol levels [28]. As far as we know, music intervention does not pose any risks or side‐effects, thus making it a safe intervention. However, even if clear evidence on effectiveness is obtained, implementation of new interventions in healthcare practice is known to be challenging [29, 30, 31]. Regarding perioperative music intervention, recently performed implementation studies achieved penetration rates of 36%–53% [32, 33, 34]. This was caused by several barriers for implementation which were not overcome before implementation. Therefore, knowing and actively addressing these barriers in advance seems essential for a successful implementation.

The IMPROVE (IMPlementation of music inteRvention in periOperative standard carE) study was set up to achieve a sustainable implementation of music intervention in perioperative standard of care in a systematic manner [35]. This study aimed to identify the barriers and facilitators for implementing perioperative music intervention in colorectal surgery.

## METHODS

### Study design

The IMPROVE study was a prospective monocentre study carried out in the IJsselland Hospital, Capelle aan den IJssel, the Netherlands. The study protocol was approved by the Medical Ethics Committee of the Erasmus University Medical Centre (registration number MEC‐2019‐0563). Implementation was carried out according to the Consolidated Framework for Implementation Research (CFIR) [36] in three steps: (1) assessment of facilitators and barriers for implementing perioperative music intervention, (2) development of a tailored implementation strategy and (3) the implementation process. As mentioned before, this paper addresses the first step only. The complete study phase overview is depicted in Appendix S1 and was extensively described in the previously published protocol paper [35].

### Study population

All patients who underwent a surgical procedure for IBD or colorectal cancer (CRC) were informed regarding the study, surveyed and written informed consent was acquired, according to the Dutch Personal Data Protection Regulation, for anxiety assessment and data collection from the patients’ medical record. Healthcare professionals involved in the perioperative process, including general surgeons, anaesthesiologists, gastrointestinal (GI) surgery ward and anaesthetist nurses, specialized nurses in GI oncology (nurse practitioner [NP]/physician assistant [PA]) and stoma care, and surgery and anaesthesiology residents, were also surveyed in this phase. A dedicated team of healthcare professionals was assembled at the implementation site. The dedicated team consisted of one person from each group of professionals, the dedicated team actively engaged in the study to reach effective implementation of the intervention.

### Outcomes

Preoperative and postoperative anxiety scores were assessed on the day of inclusion and postoperative day (POD) 1, using the visual analogue scale for anxiety (VAS‐A) on a scale of zero to 10 which is a reliable tool for assessing anxiety in the perioperative setting [37, 38]. Postoperative pain scores (numerical rating scale, NRS) were extracted from the patient's medical health record until POD 14. Assessment of facilitators and barriers for implementing perioperative music intervention in the standard of care was evaluated by using qualitative research methods in the form of surveys.

### Survey development and assessment

Two different surveys, for patients (Appendix S2) and healthcare professionals (Appendix S3), were developed. Both surveys were based on four of the five domains of the CFIR: (1) inner setting, (2) outer setting, (3) characteristics of the patients and healthcare professionals and (4) intervention. The fifth domain is the implementation process itself, which was beyond the scope of this study [36]. The patient survey (17 questions) assessed patient needs (outer setting) and characteristics of the individuals, including demographic data. Patients were questioned on POD 1 specifically about their preferences, beliefs and opinions regarding the use of recorded music in the perioperative setting and were asked to score their anxiety levels in the preoperative and postoperative period.

The healthcare professional survey (37 questions) assessed all four domains of the CFIR as previously described. This survey focused on demographic characteristics, current practices and beliefs regarding perioperative anxiety and pain management, characteristics of the site (e.g., communication, schooling, leadership etc.) and characteristics regarding the intervention itself (e.g., easy to implement etc.). The healthcare professional survey was built in an anonymous electronic survey program. A link of the survey was sent via email to members of the dedicated team, who then further distributed the survey among all professionals involved. Reminders were sent via email every month during a 7‐month period (from December 2019 until July 2020). Both surveys contained questions on current knowledge regarding the effects of music in a perioperative setting.

To ensure content validity of the surveys, the first drafts were carefully revised by members of the research team (EK/BS/ ). After consensus was reached, the survey was presented to the dedicated team for input. Any valuable comments were incorporated in the last version of the survey before it was distributed amongst healthcare professionals and patients.

### Sample size

A minimum reduction of 12 mm on VAS for pain (corresponding to an NRS of 1.2) has been shown as clinically relevant [39]. To measure the impact of music intervention on postoperative pain we performed a sample size calculation based on previous reported mean pain scores and standard deviations after colorectal surgery in the papers of Kamiński et al. and Mouawad et al. [40, 41]. We used the mean pain scores measured with VAS of patients on the first POD using patient controlled analgesia (*N* = 173, VAS = 4.6 ± 2.0) which yielded the largest sample size. To obtain a power of 80%, an alpha of 5% (*P* = 0.05), planning two‐sided testing and a drop‐out rate of 10%, a sample size of 50 patients pre‐implementation and 50 patients post‐implementation is required.

### Data analysis

The response rate was defined as the percentage of all returned surveys. Questionnaires without complete knowledge assessment were excluded from analysis. Descriptive statistics were used for outcomes of the current practices regarding anxiety and pain. Continuous data were presented using median and interquartile range (IQR). The Kruskal–Wallis test was used to analyse differences between groups. Categorical data were analysed using the chi‐squared test and Fisher's exact test if at least one group sample was less than five. Ordinal data (e.g., age and working experience) were analysed using the Kruskal–Wallis test. The Bonferroni correction was applied in the case of multiple testing in the post hoc tests. Knowledge regarding music intervention was presented by calculating a median knowledge score (MKS, IQR). For every knowledge question, a knowledge score was assessed, which ranged from 0% to 100%. The MKS was assessed by calculating the median of the MKS of all respondents.

The definitions of facilitators and barriers were based on expert opinion (research team including an implementation expert (EI)), and were expressed in percentages. An MKS under 70.0% was defined as a barrier, 70.0% or higher as a facilitator. For other barriers and facilitators a cut‐off point of 50.0% was used [42]. Data were analysed using R studio version 4.0.0.

## RESULTS

### Patients

#### Demographics

The median age of participants was 62.5 (IQR 21.8) years. The groups of patients being operated for IBD or CRC differed significantly regarding both age (*P* < 0.001) and educational level (*P* = 0.03).

#### Survey results

The complete survey results can be found in Appendix S2. In total, 50 patients were included, who all completed the survey. The majority of respondents had CRC (64.0%). IBD was the second most prevalent indication, with 20.0% of patients diagnosed with Crohn's disease and 8.0% with ulcerative colitis. The other 8.0% included colectomy based on constipation due to morbus Sjögren, connective tissue disease and diverticulitis.

#### Patients’ characteristics

The MKS was 42.9% (26.4). Educational level and reason for surgery did not have significant influence on this outcome. Respondents scored a median of 8.0 (IQR 6.0–9.0) on the importance of music in their daily life on a scale of 0–10. Patients who listened to music more frequently rated the importance of music higher (*P* = 0.05) in the post hoc test. The median age of the patients choosing classical music was significantly higher (*P* < 0.001) than patients who did not choose classical music and for pop music significantly lower (*P* < 0.001) than patients who did not choose pop music. The respondents who would like to receive music also had a significantly higher MKS compared to the patients who did not (*P* < 0.001 for all perioperative moments). Table 1 gives an overview of the facilitators and barriers found based on the surveys.

**TABLE 1:** Barriers and facilitators

Healthcare professionals	Patients
Facilitators	
Current practices	Characteristics of the individual
Adverse events due to anxiety and pain medication	Importance of music in daily life
Outer setting	Amount of music listened to in daily life
Patient‐centred care	Willingness to be informed about the intervention
Inner setting	Willingness to receive the intervention
Shared goals	
Proper cooperation with direct and indirect colleagues	
Proper amount of education	
Proper leadership in the teams	
Proper way of decision making	
Proper learning climate	
Proper room for reflection and evaluation	
Characteristics of the individual	
Need for alternatives for pain and anxiety medication	
Awareness of the effects of the intervention	
Belief in the intervention	
Belief that the intervention fits the current healthcare system	
Preference of music above medication	
Belief in own abilities for implementing	
Stable teams	
Barriers	
Current practices	Characteristics of the individual
Need for preoperative anxiety and pain medication	Knowledge regarding the intervention
Inner setting	Awareness of the use of music for patients
Way of decision making among ward nurses and nurse anaesthetists	Willingness for receiving the intervention intra‐operatively (lack of knowledge)
Room for reflection and evaluation among ward nurses and nurse anaesthetists	
Goals among nurse anaesthetists	
Cooperation with indirect colleagues among nurse anaesthetists	
Leadership among nurse anaesthetists	
Characteristics of the individual	
Knowledge regarding the intervention	

#### Perioperative anxiety and pain

No significant differences were found in anxiety scores preoperatively (*P* = 0.7) or postoperatively (*P* = 0.2) between CRC and IBD patients. For 29 patients preoperative pain scores of the morning before surgery were available; the median was zero (IQR 0–0) and did not differ significantly between CRC and IBD patients. The postoperative pain score (NRS 0–10) on POD 1 was 2.8 (IQR 2.0–3.7). When comparing groups based on diagnosis, significant differences were found between CRC and IBD patients (post hoc test; *P* = 0.03). Patients with IBD (3.4, IQR 3.0–3.7) had significantly higher pain scores compared to CRC (2.3, IQR 1.5–3.5) patients.

### Healthcare professionals

#### Demographics

The health professional groups differed significantly from each other regarding age (*P* < 0.001), work experience (*P* = 0.01) and workload (*P* < 0.001). The Dunn post hoc test with Bonferroni correction showed that nurses and physicians were older, *P* < 0.001 and *P* < 0.001 respectively, and had more working experience, *P* = 0.008 and *P* = 0.05 respectively, than residents. Physicians (*P* < 0.001) and residents (*P* < 0.001) worked significantly more hours than nurses.

#### Survey results

The complete survey results can be found in Appendix S3. The response rate was 68.3%. In all, 85.5% (59/69) of the surveys were filled in completely and 14.5% (10/69) partly. The majority of the respondents were nurses (NPs/PAs, nurse anaesthetists and GI ward nurses), 43.5% (30/69); 32% (22/69) were physicians (surgeons and anaesthesiologists) and 23.0% (16/69) were residents of the surgery and anaesthesiology departments. Within the different groups of healthcare professionals, 84.4% of the medical doctors returned the survey; this included 78.6% of the physicians and 94.1% of the residents. Among the nurses, 70.4% of the ward nurses and NPs filled in the survey and 40.0% of the nurse anaesthetists. Table 1 gives an overview of the facilitators and barriers found based on the surveys.

#### Current practices

Most (92.3%, *n* = 60) respondents were aware of the effects of music on surgical patients. Overall, 48.4% (31/64) of respondents reported additional postoperative pain medication to be requested by >50.0% of their patients (57.1%, 60.0%, 57.0% by the anaesthesiologists, nurse anaesthetists and surgeons respectively).

#### Inner setting

Fifty‐three per cent of the ward nurses and 50.0% of anaesthetist nurses stated that decision making on work processes needs to be amended. Forty‐six per cent (6/13) of the anaesthesiologists reported high variability in the teams on the GI surgery ward. This impression was confirmed by 40% of the nurse anaesthetists. Significant differences were found between the different healthcare professional groups (*P* = 0.008) for these factors. Fifty per cent of the nurse anaesthetists and NPs/PAs stated that the purposes/goals in their team differed between members. Seventy‐eight per cent (46/59) of the respondents, however, did report proper collaboration with colleagues (indirect) in other departments. The majority of the respondents stated that education regarding current guidelines only took place when guidelines were updated or newly implemented. Nevertheless, 77.6% (45/58) stated that this was sufficient. This statement was made by >50% in all groups, except by the NPs/PAs. Twenty‐five per cent of the nurses and 50.0% of the nurse anaesthetists did not agree on the statement that the learning climate is safe and/or stimulating.

#### Outer setting

Regarding patient needs, almost all respondents would apply music if this was the patient's choice (91.9%, 57/62). Regarding peer pressure, 87.7% believed that hospitals should follow similar general policies/protocols for their patients.

#### Characteristics of the intervention and individuals

The MKS of all respondents was 58.3% (IQR 53.8–64.3), which did not differ between the groups (*P* = 0.198). Overall, respondents believed that there was a need for an alternative to pharmacological interventions reducing perioperative anxiety and pain, believed in the effectiveness of perioperative music intervention and believed that music should be part of standard care. Most of the respondents were already informed on the effects of music (92.3%, 60/65). This existing awareness of the effects of music did not affect the MKS (*P* = 0.40). Respondents scored their confidence/ability in successfully applying music intervention with a median of 8.0 (IQR 7.0–8.0); no significant differences were found between healthcare professionals (nurse vs. resident vs. physician; *P* = 0.184). Table 2 shows what healthcare professionals require, mainly equipment and instructions, in order to successfully ensure implementation of perioperative music.

**TABLE 2:** Requirement for implementation

Function	Equipment	Instructions	Clinical lessons	Exemplary behaviour	Culture change	Time	Feedback
Anaesthesiologists	×	×					
Surgeons	×	×	×	×			
Department nurses	×	×	×				
Nurse anaesthetists	×	×					
NPs/PAs	×	×	×	×	×	×	
Resident surgery	×	×			×	×	
Resident anaesthesiology	×	×	×	×	×	×	×

Abbreviations: NP, nurse practitioner; PA, physician assistant.

## DISCUSSION

This is the first study assessing barriers and facilitators for implementing preoperative, intra‐operative and postoperative music intervention during hospital admission through surveys of postoperative patients and healthcare professionals (Appendix S4). Furthermore, anxiety and pain in the perioperative phase before implementation were also evaluated.

A reasonably high response rate in healthcare professionals (68.3%) was achieved, and therefore it can be assumed that the survey results are reliable for this setting (perioperative colorectal surgical standard of care in the IJsselland Hospital, Capelle aan den IJssel, the Netherlands) [42, 43]. Several important facilitators were identified for patients, which include the importance of music in daily life, the number of hours listening to music in daily life and the willingness to be informed about and receive the music intervention during (future) hospital admission. For the healthcare professionals all domains of the CFIR were considered to be facilitatory for implementation. The demographics of the healthcare professionals show that a larger group of the perioperative healthcare professionals encompass nurses and physicians with higher age and (consequently) more working experience. This suggests that the site for implementation is mature and therefore implementation of a new intervention should be feasible, as the maturity of a site is an important factor for implementation [44, 45]. Several barriers were found, including lack of knowledge of the intervention for all healthcare professional groups (nurses, physicians, residents). The inner setting of nurses’ and nurse anaesthetists’ barriers consisted of the current way decisions were made and insufficient time for reflection and evaluation. Furthermore, nurse anaesthetists’ experience is that there is no proper leadership, that there are differences in thoughts and goals between members of the team, and collaboration with other departments is not optimal. Amongst patients, barriers only consisted of lack of knowledge about the intervention. Finally, preoperative and postoperative anxiety and pain scores were assessed since these could be barriers (if low) or facilitators (if high) for implementation. Moderate perioperative anxiety levels and both preoperative and postoperative moderate pain scores were found for all patients with the provided postoperative pain management. Since the preoperative anxiety level was assessed postoperatively this anxiety level is subject to recall bias. The perioperative anxiety and pain scores may be influenced by several factors. Important factors that may play a role are the way patients are informed preoperatively regarding the perioperative procedure. If counselling is carried out adequately this may lead to reduction in preoperative anxiety experienced by the patients [46]. Furthermore, the use of standardized postoperative opioid regimens leads to more controlled pain experiences in patients. In the final step of the IMPROVE study assessment of the effects of the music intervention on medication use for anxiety and pain will be carried out.

This first phase of the IMPROVE study was to identify determinants which may influence the process of implementing music intervention as an alternative and/or supplement to reducing perioperative anxiety and pain. By identifying the facilitators and barriers, a tailored implementation strategy to achieve swift implementation and proper adherence to the intervention in clinical practice can be conducted. The implementation strategy will be based on the Expert Recommendations for Implementing Change (ERIC) [47]. Based on the ERIC, the proper mechanism of action will be selected separately for each strategy, since it is known that tailored multilevel strategies are more likely to be effective than single strategies [45]. For example, in this phase it was found that the knowledge regarding music intervention in healthcare is low; this is considered a barrier for implementation. The implementation strategy for this would be developing and distributing educational materials, for example pocket cards for nurses with information about how to apply the intervention, in order to handle this barrier [35]. Finally, the implementation strategies will be operationalized by using the seven dimensions proposed by Proctor et al. [48]. A guideline to improve the reporting of implementation strategies in research studies and to stimulate further identification of elements pertinent to implementation strategies is proposed by the authors.

### Strengths and limitations

The most important strength of this study is that it assessed barriers and facilitators according to one of the most widely used frameworks for implementation. Second, apart from the healthcare professionals’ perspectives, patients’ perspectives were also assessed. Lastly, the surveys developed for this study make it possible to identify facilitators and barriers in other settings. Nevertheless, several limitations must be considered. This study is conducted in one specific surgical population and in one regional teaching hospital in the Netherlands. The identified facilitators and barriers may therefore not be completely applicable to other settings (e.g., psychiatrics population, tertiary referral hospital) and interventions. However, the surveys developed for this study can be used to detect facilitators and barriers in other settings. Furthermore, the number of patients included is low, questioning the validity of the survey results. As for all surveys, almost one‐third of the healthcare professionals did not respond which suggests that selection bias cannot be ruled out.

## CONCLUSION

In this first step of the IMPROVE study several important facilitators and barriers for implementing music intervention in the perioperative standard of care were identified, in which the facilitating factors were more prominent for a specific setting. The most important barriers for implementation were lack of knowledge regarding the intervention in both patients and healthcare professionals and the professional climate and culture factors among the nurses. Overall, the attitudes, perceptions, beliefs of patients and healthcare professionals regarding music intervention were facilitatory and therefore successful implementation is expected. Lastly, the surveys developed for this study can be used as a tool to identify facilitators and barriers in other settings.

## CONFLICT OF INTEREST

None declared.

## AUTHOR CONTRIBUTIONS

JJ conceived the study idea. EK, EI and BS coordinated the study. EK, OR, BS, BH, EG, EI, JL, JJ and MK interpreted the data. EK, OR, BS and EI wrote the first draft of the manuscript. EK, OR, BS, BH, EG, EI, JL, JJ and MK critically revised the manuscript. EK, OR, BS, BH, EG, EI, JL, JJ and MK had full access to all the data in the study and can take responsibility for the integrity of the data and the accuracy of the data analysis.

## ETHICAL APPROVAL

The study protocol was approved by the Medical Ethics Committee of the Erasmus University Medical Centre (registration number MEC‐2019–0563).

## PREREGISTRATION OF STUDY OR ANALYSIS PLAN DISCLOSURE

No preregistration exists for the studies reported in this paper.

## Supporting information

Appendix S1

Appendix S2

Appendix S3

Appendix S4 Visual Abstract

## Data Availability

The data that support the findings of this study are available on request from the corresponding author. The data are not publicly available due to privacy or ethical restrictions.
