# Supplementary material for: Implementation of music in colorectal perioperative standard care—barriers and facilitators among patients and healthcare professionals
Source: Colorectal Dis. 2022 Apr 6;24(7):868–75. doi: 10.1111/codi.16102 (PMC9544166; doi:10.1111/codi.16102)
Supplement: Supplementary file 1 — Appendix S1 [file CODI-24-868-s001.docx]

Appendix 1. Study phase overview

**Implementation strategy**

**Phase 3**

**Phase 2**

**Phase 1**

1. Exposure to intervention
2. Experiences
3. Adjustments
4. Informing patients
5. Music application
6. Data collection

**Process evaluation**

**Implementation**

1. **Characteristics of individuals:**

Demographics

Knowledge

Attitudes

Opinions

1. **Data collection**
2. **Characteristics of the intervention**
3. **Current practices**
4. **Outer setting**
5. **Inner setting**
6. **Characteristics of individuals**

**Health care professional**

**Patient**

**Multifaceted strategy**

1. **Mechanism of action:**

Expert Recommendations for Implementing Change (ERIC) strategies

1. **Operationalization:**
2. Actor
3. Action
4. Target
5. Temporality
6. Dose
7. Implementation outcomes
8. Justification

**Implementation process**

**Assessment current practices and barriers and facilitators**
