# Supplementary material for: Implementation of music in colorectal perioperative standard care—barriers and facilitators among patients and healthcare professionals
Source: Colorectal Dis. 2022 Apr 6;24(7):868–75. doi: 10.1111/codi.16102 (PMC9544166; doi:10.1111/codi.16102)
Supplement: Supplementary file 2 — Appendix S2 [file CODI-24-868-s002.docx]

**Appendix 2. Patient survey and results**

**Survey**

1. What is the highest education level you achieved? (choose one of the options below)

□ Primary school □ MBO 12

□ VMBO/MAVO1 □ MBO 22

□ HAVO1 □ MBO 32

□ VWO1 □ MBO 42

□ HBO2 □ University

*^1^These are Dutch equivalents of high school, with VMBO/MAVO being the lowest and VWO the highest level. HAVO rests in between.*

*^2^These are Dutch equivalents of college, with MBO being the lowest (1-4 corresponds with ascending difficulty) and HBO the highest level.*

2. Music affects the following factors in patients:

(multiple answers possible)

□ Anxiety

□ Surgery length

□ Pain

□ Nausea

□ Stress

□ Vomiting

□ Pain medication use

□ Complication risk

□ I do not know

3. Music possibly affects the following factors in operators: (multiple answers possible)

□ Stress

□ Speed

□ Quality

□ Attention

□ Precision

□ Communication

□ I do not know

4. At what moment is music effective when the patient listens to it?

(multiple answers possible)

□ Before surgery

□ During surgery

□ After surgery

□ I do not know

5. Statement: Relatively, music does not know risks when compared to other treatments for pain and anxiety.

(choose one of the options below)

□ True □ False □ I do not know

6. How important is music for you in your daily life? Choose a number between 0 and 10, considering 0 = not important at all and 10 = very important.

□ □ □ □ □ □ □ □ □ □ □

0 1 2 3 4 5 6 7 8 9 10

7. What is your favorite musical genre? (multiple answers possible)

□ Blues □ Hip Hop □ Classical □ Rhythm and Blues (R&B)

□ Country □ Jazz □ Metal □ Pop

□ Rock □ Other

8. How often do you listen to music?

□ Several hours per day □ Several hours per week

□ Sometimes □ Rarely

9. What is the most suitable device for you to listen to music before, during and after surgery?

Before surgery During surgery After surgery

Headphones □ □ □

Earbuds □ □ □

CD-player □ □ □

Other □ also answer question 9a □ also answer question 9b □ also answer question 9c

“I do not know” □ □ □

9a. Specify what device for applying music before surgery you have in mind.

9b. Specify what device for applying music during surgery you have in mind.

9c. Specify what device for applying music after surgery you have in mind.

10. Choose what holds your preference.

□ I think I should bring the music and music equipment with me to the hospital myself.

□ I think the hospital should have music and music equipment available for its patients.

11. Would you like to be informed about the effects of music in healthcare?

□ Yes □ No

12. If you knew that music causes positive effects in patients that undergo a surgical procedure, would you then like to listen to the music of your preference through headphones/earbuds?

□ Yes □ No

13a. If available, would you like to listen to the music of you preference through headphones/earbuds preoperatively?

□ Yes □ No

13b. If available, would you like to listen to the music of you preference through headphones/earbuds intraoperatively?

□ Yes □ No

13c. If available, would you like to listen to the music of you preference through headphones/earbuds postoperatively?

□ Yes □ No

14. Are you familiar with the application of music intervention through headphones/earbuds for patients in the current health care?

□ Yes □ No

15. When would you like to be informed about the use of music intervention perioperatively?

□ During explanation about the surgical procedure (surgeon)

□ During explanation about the narcosis (anesthesiologist)

□ During the appointment about you colostomy bag

□ During the appointment regarding your problems/worries

□ On the day of hospital admission

□ On the day of surgery

16. Choose what holds your preference

□ I would rather listen to my own music during hospital admission

□ Music lists, which I can choose from, should be offered by the hospital

17a. How much anxiety did you experience just before the surgical procedure? Choose a number between 0 and 10, considering 0 = not anxious at all and 10 = very anxious.

□ □ □ □ □ □ □ □ □ □ □

0 1 2 3 4 5 6 7 8 9 10

17b. How much anxiety did you experience after the surgical procedure? Choose a number between 0 and 10, considering 0 = not anxious at all and 10 = very anxious.

□ □ □ □ □ □ □ □ □ □ □

0 1 2 3 4 5 6 7 8 9 10

If you wish to discuss the results of the survey in an interview, you can leave your email address below: ………………………………………………………………………………………………………

**Results**

| Total respondents | N  50 | %  100.0 |
| --- | --- | --- |
| Demographics |  |  |
| Operation  Oncological  IBD  Other | N  32  14  4 | %  64.0  28.0  8.0 |
| Age*  Overall  Oncological  IBD  Other  **Kruskal- Wallis p <0.001* | Median  62.5  70.5  41.5  54.0 | IQR  21.8  10.3  27.5  6.5 |
| Education level*  Primary school or less  High school  College  **Kruskal- Wallis p <0.001* | N  6  22  22 | %  12.0  44.0  44.0 |
| Characteristics of the individuals |  |  |
| MKS  Overall  Oncological  IBD  Other | Median  42.9  42.9  33.2  52.3 | IQR  26.4  20.7  33.6  6.3 |
| How important is music for you in your daily life?  (NRS 0-10, 0 = not important at all, 10 = very important)  Total respondents  Oncological  IBD  Other | N  48  30  14  4 | Median (IQR)  8.0 (3.0)  8.0 (1.8)  7.5 (2.0)  7.0 (0.8) |
| What is your favorite music genre? (Multiple answers possible)  Total respondents  Blues  Hip Hop  Classic  Rhythm and Blues (R&B)  Country  Jazz  Metal  Pop  Rock  Other  Dutch music  Pop ’60-‘80  Radio music  Church music  Dance  Soul  Trance, Hardstyle, Easy listening, French chansons, Motown big bang,  Reiki music    Yoga, Indie pop | N  50  9  6  22  11  13  11  0  23  5  24  6  4  3  2  2  2  1 per genre  2 | %  100.0  18.0  12.0  44.0  22.0  26.0  22.0  0.0  46.0  10.0  48.0 |
| How often do you listen to music?  Total respondents  Several hours per day  Several hours per week  Sometimes  Rarely | N  50  37  9  3  1 | %  100.0  74.0  18.0  6.0  2.0 |
| What would be the best device to use to listen to music preoperatively?  Total respondents  Headphone  Earbuds  CD-player  I do not know  Other  Radio  None | N  49  23  16  2  5  3  2  1 | %  98.0  46.9  30.6  4.1  10.2  8.2 |
| What would be the best device to use to listen to music intraoperatively?  Total respondents  Headphone  Earbuds  CD-player  I do not know  Other  None  Radio | N  37  16  8  4  6  3  2  1 | %  74.0  43.2  21.6  10.8  16.2  8.1 |
| What would be the best device to use to listen to music postoperatively?  Total respondents  Headphone  Earbuds  CD-player  I do not know  Other  Headphones or earbuds  Radio | N  47  23  15  3  4  2  1  1 | %  94.0  48.9  31.9  6.4  8.5  4.3 |
| If music were to become part of the standard healthcare, who should arrange for the music equipment?  Total respondents  The hospital  The patient | N  47  31  16 | %  94.0  66.0  34.0 |
| Would you like to be informed about the effects of music in healthcare?  Total respondents  Yes  No | N  49  31  18 | %  98.0  63.3  36.7 |
| If you knew music had positive effects on patients undergoing surgery, would you then like to listen to music of your preference?  Total respondents  Yes  No | N  48  45  3 | %  96.0  93.8  6.3 |
| If available, would you like to listen to music of your preference before your surgical procedure?  Total respondents  Yes  No | N  50  43  7 | %  100.0  86.0  14.0 |
| If available, would you like to listen to music of your preference during your surgical procedure?  Total respondents  Yes  No | N  48  22  26 | %  96.0  45.8  54.2 |
| If available, would you like to listen to music of your preference after your surgical procedure?  Total respondents  Yes  No | N  49  41  8 | %  98.0  83.7  16.3 |
| Are you familiar with the application of music in healthcare?  Total respondents  Yes  No | N  50 13  37 | %  100.0  26.0  74.0 |
| When would you like to be informed about the use of music around your surgical procedure?  Total respondents  During information about the surgery (surgeon)  During information about the narcosis (anesthesiologist)  During the appointment regarding the colostomy bag  During the appointment regarding your problems/worries  On the day of hospital admission  On the day of surgery  Other  On multiple occasions  During conversation with the nurse  On the day of admission and the day of surgery  During narcosis information and on the day of admission  Through an information folder | N  50  13  9  1  0  14  3  10  6  1  1  1  1 | %  100.0  26.0  18.0  2.0  0.0  28.0  6.0  20.0 |
| Which of the following holds your preference?  Total respondents  Listening to my own music during my hospital admission  Listening to music lists, from which I can choose, composed by the hospital | N  46  24  22 | %  92.0  52.2  47.8 |
