# Supplementary material for: Implementation of music in colorectal perioperative standard care—barriers and facilitators among patients and healthcare professionals
Source: Colorectal Dis. 2022 Apr 6;24(7):868–75. doi: 10.1111/codi.16102 (PMC9544166; doi:10.1111/codi.16102)
Supplement: Supplementary file 3 — Appendix S3 [file CODI-24-868-s003.docx]

**Appendix 3. Health care professional survey and results**

**Survey**

1. What is your function within the hospital?

(choose one of the options below)

□ Vascular surgeon

□ Trauma surgeon

□ Gastro-intestinal surgeon

□ Anesthesiologist

□ Surgery resident

□ Anesthesiology resident

□ Surgery intern

□ Anesthesiology intern

□ Anesthetist nurse

□ Nurse specialist

□ Physician assistant

□ Nurse

□ I am a student

□ Other; …………………………………………………………

2. How many years of work experience do you have in your current function?

(choose one of the options below)

□ <1 year □ 1-4 years □ 5-9 years □ 10-20 years □ >20 years

3. How many hours do you work per week?

(choose one of the options below)

□ <12 hours □ 12-20 hours □ 21-28 hours □ 29-36 hours □ >36 hours

4. How old are you?

(choose one of the options below)

□ <25 years □ 25-34 years □ 35-44 years □ 45-54 years □ >54 years

5. Music affects the following factors in patients:

(multiple answers possible)

□ Anxiety

□ Surgery length

□ Pain

□ Nausea

□ Stress

□ Vomiting

□ Pain medication use

□ Complication risk

□ I do not know

6. Music possibly affects the following factors in operators:

(multiple answers possible)

□ Stress

□ Speed

□ Quality

□ Attention

□ Precision

□ Communication

□ I do not know

7. At what moment is music effective when the patient listens to it?

(multiple answers possible)

□ Before surgery

□ During surgery

□ After surgery

□ I do not know

8. Statement: Relatively, music does not know risks when compared to other treatments for pain and anxiety.

(choose one of the options below)

□ True □ False □ I do not know

9. What percentage of your patients do you think requests additional pain medication after surgery?

□ Less than 10%

□ 10-25%

□ 26-50%

□ 51-75%

□ More than 75%

□ I do not know

10. What percentage of your patients do you think requests/receives an intervention for anxiety/nerves before surgery?

□ Less than 10%

□ 10-25%

□ 26-50%

□ 51-75%

□ More than 75%

□ I do not know

11. What percentage of your patients do you think have side-effects due to pain- or anxiety medication?

□ Less than 10%

□ 10-25%

□ 26-50%

□ 51-75%

□ More than 75%

□ I do not know

12. Do you think there should be an alternative to pharmaceutical treatment of perioperative pain and anxiety?

□ Yes □ No

13. Is music applied for patients in the hospital?

□ Yes (proceed to question 13a)

□ No (you can skip question 13a)

□ I do not know (you can skip question 13a)

13a. Where in the hospital is music applied?

(multiple answers possible)

□ In the operating room

□ At the nursery department

□ At the intensive care

□ Other; …………………………………………………………………………………

14. Did you hear about the effects of music in surgical patients?

□ Yes (proceed to question 14a) □ No (you can skip question 14a)

14a. Please indicate through which medium you heard about the effects of music in surgical patients. (multiple answers possible)

□ Colleague

□ Presentation and/or clinical lesson

□ Television

□ Newspaper

□ Radio

□ Other; ……………………………………..

15. Do you believe in music as an alternative to pain- and anxiety treatment?

□ Yes □ No □ I do not know

16a. Do you think music intervention (recorded) should be made part of the perioperative standard care?

□ Yes □ No

16b. Would you like to be informed about the effects of and the scientific evidence about music in the perioperative setting?

□ Yes □ No

17. If recorded music and medication would be equally effective regarding pain- and anxiety treatment, what would be your preference?

□ Music □ Medication □ Music and Medication □ I do not know

18. What do you think is the most suitable device for listening to music before, during and after surgery

Before surgery During surgery After surgery

Headphones □ □ □

Earbuds □ □ □

CD-player □ □ □

Other □ also answer question 18a □ also answer question 18b □ also answer question 18c

“I do not know” □ □ □

18a. Specify what device for applying music before surgery you have in mind.

18b. Specify what device for applying music during surgery you have in mind.

18c. Specify what device for applying music after surgery you have in mind.

19. If music has positive effect, the patient should;

□ Be free in choosing his/her preferred music type

□ Listen to music selections, based on literature

20. If music were to become part of standard care, who should arrange the music equipment?

□ The patient

□ The hospital

□ Other;……………………………………………………………………………………………….

21. If you would not support the music intervention, but it is the patients’ preference, would you apply the intervention?

□ Yes Provide your comments here:

□ No

□ I do not know

22. Does application of recorded music in patients fit in the current care at your department?

□ Yes □ No □ I do not know

23. Do you think application of recorded music is easy to implement in standard care?

□ Yes Provide your comments here:

□ No

□ I do not know

24. Do you think you will succeed in adequately applying music intervention in patients?

Select a number between 0 and 10, with 0 meaning ‘I will not succeed at all’ and 10 meaning ‘I will definitely succeed’

□ □ □ □ □ □ □ □ □ □ □

0 1 2 3 4 5 6 7 8 9 10

25. Implementation of recorded music before, during and after surgery require;

□ Adjustments in my work methods, with large impact

□ Adjustments in my work methods, with slight impact

□ Adjustments in my work methods, without impact

26. What do your colleagues need to ensure successful implementation of music intervention?

(multiple answers possible)

□ Instructions

□ Clinical lessons

□ Facilities (e.g. music equipment)

□ Exemplary behavior

□ Feedback on practice

□ Culture change

□ Time

□ I do not know

□ Other; …………………………………………………………………………………………………

27. Are there set teams / colleagues on the gastro-intestinal (GI) surgery department?

□ Always □ Often □ Rarely □ Never □ I do not know

28. Who makes important decisions (e.g. regarding department organization) at your department?

□ Few persons □ A certain group □ Collectively (all involved individuals)

29. The manner in which decisions are made in my team / at my department is;

□ Good □ Could be better □ Bad

30. Collaboration with my direct colleagues is;

□ Good □ Could be better □ Bad

31. In general, I experience the purposes and goals of my team as;

□ Different □ Shared

32. Collaboration with other departments /specialists (e.g. GI-surgery department, anesthesiologists, surgeons) is;

□ Decent □ Could be better □ Bad

33. How often are lessons on current guideline / protocol adherence given?

□ Only if something new is introduced/ implemented

□ Weekly

□ Monthly

□ Once in three months

□ Once a year

33a. Is this sufficient for you?

□ Yes (you can skip question 7b)

□ No (proceed to question 7b)

33b. If not, what could be a solution for this?

34. The learning climate (e.g. the possibility to ask questions and receive feedback) is safe and stimulating;

□ Strongly agree □ Agree □ Neutral □ Disagree □ Strongly agree

35. Enough time for reflection and evaluation is present;

□ Strongly agree □ Agree □ Neutral □ Disagree □ Strongly agree

36. Good leadership within the team / at the department / in the operating room is present;

□ Strongly agree □ Agree □ Neutral □ Disagree □ Strongly agree

37. Do you think it is important that hospitals in the Netherlands follow a share general policy?

□ Yes □ No

If you wish to discuss the results of the survey in an interview, you can leave your email address below:

……………………………………………………………………………………………………….

*The inner setting includes the assessment of characteristics of the hospital/implementation site, including network (logistics) and the way of intercommunication, culture (norms and values), level of knowledge, and implementation climate (tension for change, feedback, learning climate etc.). The outer setting includes assessment of patient needs and peer pressure (e.g. need hospitals to have the same policies).

**Results**

| Total respondents | | N  69 | %  68.3 |
| --- | --- | --- | --- |
| Demographics | |  |  |
| Function  Nurse  Physician  Resident | | 68  30  22  16 | 67.3  44.1  31.9  23.2 |
| Age*  Nurse  Physician  Resident  **Kruskal-Wallis p <0.001 (between function)* | 25-34 years  35-44 years  45-54 years  >54 years  25-34 years  35-44 years  45-54 years  >54 years  25-34 years  35-44 years | 64  9  4  5  8  1  12  8  1  14  1 | 63.4  34.6  15.4  19.2  30.8  4.8  57.1  38.1  4.8  93.3  6.4 |
| Work experience*  Nurse  Physician  Resident  *Kruskal-Wallis p =0.01 (between function)* | 2-4 years  5-9 years  10-20 years  >20 years  2-4 years  5-9 years  10-20 years  >20 years  2-4 years  5-9 years | 57  8  2  7  11  3  7  8  3  5  2 | 56.4  28.6  7.1  25.0  39.3  14.3  33.3  38.1  14.3  71.4  28.6 |
| Workload*  Nurse  Physician  Resident  **Kruskal-Wallis p <0.001 (between function)* | 12-20 hours  21-28 hours  29-36 hours  >36 hours  12-20 hours  21-28 hours  29-36 hours  >36 hours  12-20 hours  21-28 hours  29-36 hours  >36 hours | 68  2  8  19  1  1  0  0  21  0  0  0  15 | 67.3  6.7  26.7  63.3  3.3  4.5  0.0  0.0  95.5  0.0  0.0  0.0  100.0 |
| Current practices |  |  |  |
| Is music intervention currently applied in the ‘IJsselland Hospital’?  Total respondents  Yes  No  “I do not know” | 65  14  34  17 | 64.4  21.5  52.3  26.2 |  |
| If yes, where in the ‘IJsselland Hospital’ is music intervention applied?  Total respondents  Operating room  Nursery department  Intensive care  Other  In research context and on patients request in the operating room | N  14  11  1  3  1 | %  78.6  7.1  21.4  7.1 |  |
| What percentage of your patients requests additional postoperative pain medication?  Total respondents  < 10%  10 – 25%  26 – 50%  51 – 75%  > 75%  “I do not know” | N  64  0  11  13  22  9  9 | %  63.4  0.0  17.2  20.3  34.4  14.1  14.1 |  |
| What percentage of your patients requests a preoperative pain- or anxiety intervention?  Total respondents  < 10%  10 – 25%  26 – 50%  51 – 75%  > 75%  “I do not know” | N  64  33  17  7  2  1  4 | %  63.4  51.6  26.6  10.9  3.1  1.6  6.2 |  |
| What percentage of your patients experiences adverse effects due to pain- or anxiety medication?  Total respondents  < 10%  10 – 25%  26 – 50%  51 – 75%  > 75%  “I do not know” | N  64  5  17  21  7  8 | %  63.4  7.8  26.2  32.8  10.9  12.5 |  |
| Outer setting | |  |  |
| If you would not support music intervention, but it is the patients’ preference, would you apply the intervention?  Total respondents  Yes  No  “I do not know” | | 62  57  0  5 | 61.4  91.9  0.0  8.1 |
| Do you think it is important that hospitals in the Netherlands follow a share general policy?  Total respondents  Yes  No | | 57  50  7 | 56.4  87.7  12.3 |
| Inner setting | |  |  |
| Are there set teams / colleagues on the gastro-intestinal (GI) surgery department?  Total respondents  Always  Often  Rarely  Never  I do not know | | 59  2  33  6  7  11 | 58.4  3.4  55.9  10.2  11.9  18.6 |
| Who makes important decisions (e.g. regarding department organization) at your department?  Total respondents  A few people  A certain group  Collectively  I do not know | | 59  8  18  18  15 | 58.4  13.6  30.5  30.5  25.4 |
| The manner in which decisions are made in my team / at my department is;  Total respondents  Good  Could be better  Bad | | 56  31  21  4 | 55.4  55.4  37.5  7.1 |
| Collaboration with my direct colleagues is;  Total respondents  Good  Could be better  Bad | | 58  52  6  0 | 57.4  89.7  10.3  0.0 |
| In general, I experience the purposes and goals of my team as;  Total respondents  Different  Shared | | 58  16  42 | 57.4  27.6  72.4 |
| Collaboration with other departments /specialists is;  Total respondents  Decent  Could be better  Bad | | 59  46  13  0 | 58.4  78.0  22.0  0.0 |
| How often are lessons on current guideline / protocol adherence given?  Total respondents  Only if something new is introduced  Weekly  Monthly  Once in three months  Once a year | | 58  31  4  12  7  4 | 57.4  53.4  6.9  20.7  12.7  6.9 |
| Is this sufficient for you?  Total respondents  Yes  No | | 58  45  13 | 57.4  77.6  22.4 |
| If not, what could be a solution for this?  Total respondents  Structural schooling (x3)  Plan more repetition (x3) | |  |  |
| The learning climate is safe and stimulating;  Total respondents  Strongly agree  Agree  Neutral  Disagree  Strongly disagree | | 58  19  28  9  2  0 | 57.4  32.8  48.3  15.5  3.4  0.0 |
| Enough time for reflection and evaluation is present;  Total respondents  Strongly agree  Agree  Neutral  Disagree  Strongly disagree | | 57  9  20  18  10  0 | 56.4  15.8  35.1  31.6  17.5  0.0 |
| Good leadership within the team / at the department / in the operating room is present;  Total respondents  Strongly agree  Agree  Neutral  Disagree  Strongly disagree | | 57  11  19  18  8  1 | 56.4  19.3  33.3  31.6  14.0  1.8 |
| Characteristics of the individuals | |  |  |
| MKS by function*  Total respondent  Physicians  Nurses  Residents  Overall  **Kruskal-Wallis p = 0.20* | | N  69  22  30  16  69 | Median(IQR)  58.3 (6.0)  61.5 (15.2)  53.4 (21.7)  58.3 (10.4) |
| Did you hear about the effects of music on surgical patients?  Total respondents  Yes  No | | N  65  60  5 | %  64.4  92.3  7.7 |
| If yes, through what medium did you hear about the effects of music on surgical patients?  Total respondents  Colleague  Presentation and / or clinical lessons  Television  Newspaper  Radio  Other  Study  Internet  Applied in the past  Multiple  Patient | | N  60  38  36  31  9  3  6  2  1  1  1  1 | %  59.4  58.5  55.4  47.7  13.8  4.6  33.3 |
| Should there be an alternative to perioperative pain- and anxiety medication?  Total respondents  Yes  No | | N  64  56  8 | %  63.4  87.5  12.5 |
| Do you believe in music intervention as an alternative to pain- and anxiety medication?  Total respondents  Yes  No  “I do not know” | | N  63  54  3  6 | %  62.4  58.7  4.8  9.5 |
| Do you think music intervention (recorded) should be part of perioperative standard care?  Total respondents  Yes  No | | 63  54  9 | 62.4  85.7  14.3 |
| Would you like to be informed about the effects of and scientific proof regarding perioperative music intervention?  Total respondents  Yes  No | | 62  54  8 | 61.4  87.1  12.9 |
| If recorded music and medication are equally effective in pain and anxiety management, which would be your preference?  Total respondents  Music  Medication  Music and medication  “I do not know” | | 62  40  0  22  0 | 61.4  64.5  0.0  35.5  0.0 |
| If music has positive effects, what is the best medium for applying music on a patient;  Preoperatively:  Total respondents  Headphone  Earbuds  CD-player  Other  One earbud  “I do not know “  Intraoperatively:  Total respondents  Headphone  Earbuds  CD-player  Other  Two earbuds  Speakers  “I don’t know”  Postoperatively:  Total respondents  Headphone  Earbuds  CD-player  Other  One earbud  “I don’t know” | | 61  41  14  1  1  4  58  33  16  4  2  1  1  3  59  37  14  3  1  5 | 60.4  67.1  23.0  1.6  1.6  6.6  57.4  56.9  27.6  6.9  2.4  5.2  58.4  61.7  23.3  5.0  1.7  8.3 |
| If music has positive effects, the patient should;  Total respondents  Be free to listen to the music type of their choice  Listen to music selections based on literature | | 63  48  15 | 62.4  76.2  23.8 |
| If music were to become part of the standard care, who should arrange the music equipment?  Total respondents  The patient  The hospital  Other  Both  The patient if he/she has the opportunity to do so | | 63  9  46  8  7  1 | 62.4  14.3  73.0  12.7 |
| Does applying music intervention on patients fit into the current care of your department?  Total respondents  Yes  No  “I do not know” | | 61  40  8  13 | 60.4  65.6  13.1  21.3 |
| Do you think applying music intervention is easy to implement in the standard care?  Total respondents  Yes  No  “I do not know” | | 63  53  1  9 | 62.4  84.1  1.6  14.3 |
| Do you think you will succeed in adequately applying music intervention on patients? (0-10, 0 = I will not succeed at all, 10 = I will definitely succeed)*  Total respondents  Physicians  Nurses  Residents  Overall  *Kruskal-wallis p = 0.184 | | N  63  22  28  13  63 | Median(IQR)  8.0(1.0)  7.0(1.0)  8.0(1.0)  8.0(1.0) |
| Implementation of recorded music before, during and after surgery require the following from me;  Total respondents  Adaptations in my work methods, with large impact  Adaptations in my work methods, with low impact  Adaptations in my work methods, without impact | | N  62  0  52  10 | %  61.4  0.0  83.9  16.1 |
| What do your colleagues need to ensure successful implementation of perioperative music intervention?  Total respondents  Instructions  Clinical lessons  Facilities  Exemplary behavior  Feedback  Change of culture  Time  “I do not know”  Other | | 63  50  29  51  25  16  25  22  3  0 | 62.4  79.4  46.0  81.0  39.7  25.4  39.7  34.9  4.7  0.0 |
