# Supplementary material for: Implementation of music in colorectal perioperative standard care—barriers and facilitators among patients and healthcare professionals
Source: Colorectal Dis. 2022 Apr 6;24(7):868–75. doi: 10.1111/codi.16102 (PMC9544166; doi:10.1111/codi.16102)
Supplement: Supplementary file 4 — Appendix S4 Visual Abstract [file CODI-24-868-s004.pptx]

## Slide 1
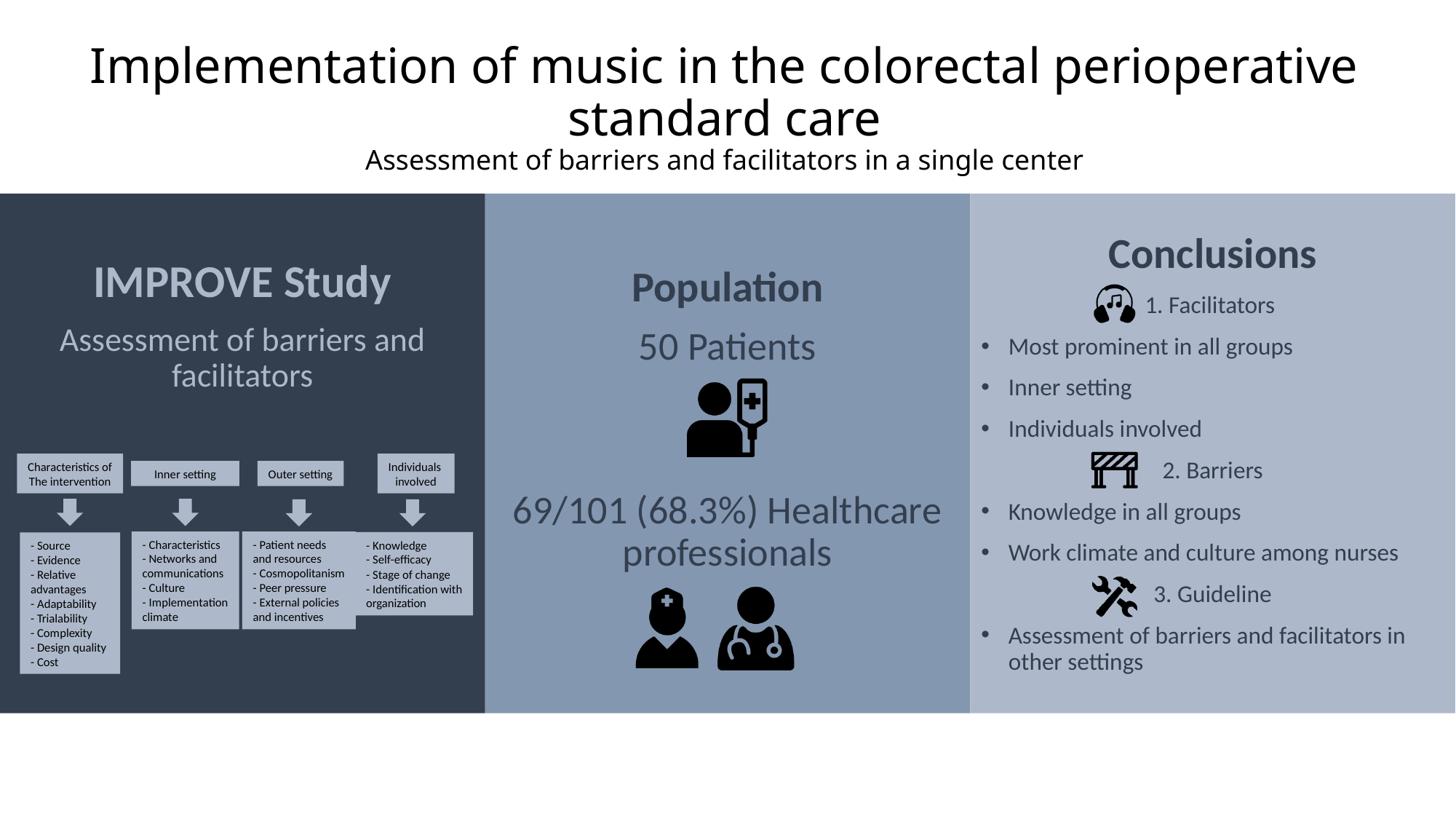

# Implementation of music in the colorectal perioperative standard care Assessment of barriers and facilitators in a single center
IMPROVE Study
Assessment of barriers and facilitators
Population
50 Patients
69/101 (68.3%) Healthcare professionals
Conclusions
1. Facilitators
Most prominent in all groups
Inner setting
Individuals involved
2. Barriers
Knowledge in all groups
Work climate and culture among nurses
3. Guideline
Assessment of barriers and facilitators in other settings
Characteristics of
The intervention
Inner setting
Outer setting
Individuals
involved
- Characteristics
- Networks and
communications
- Culture
- Implementation
climate
- Patient needs
and resources
- Cosmopolitanism
- Peer pressure
- External policies
and incentives
- Source
- Evidence
- Relative
advantages
- Adaptability
- Trialability
- Complexity
- Design quality
- Cost
- Knowledge
- Self-efficacy
- Stage of change
- Identification with
organization
